# Inhaled Sedation for Invasively Ventilated COVID-19 Patients: A Systematic Review

**DOI:** 10.3390/jcm11092500

**Published:** 2022-04-29

**Authors:** Giovanni Landoni, Olivia Belloni, Giada Russo, Alessandra Bonaccorso, Gianmarco Carà, Matthieu Jabaudon

**Affiliations:** 1 Department of Anaesthesia and Intensive Care, IRCCS San Raffaele Scientific Institute, 20132 Milan, Italy; belloni.olivia@hsr.it (O.B.); russo.giada@hsr.it (G.R.); bonaccorso.alessandra@hsr.it (A.B.); cara.gianmarco@hsr.it (G.C.); 2 School of Medicine, Vita-Salute San Raffaele University, 20132 Milan, Italy; 3 Department of Perioperative Medicine, CHU Clermont-Ferrand, F-63000 Clermont-Ferrand, France; mjabaudon@chu-clermontferrand.fr; 4 GReD, Université Clermont Auvergne, CNRS, INSERM, F-63000 Clermont-Ferrand, France

**Keywords:** volatile anesthetics, coronavirus disease 2019, acute respiratory distress syndrome, critical care, deep sedation, volatile anesthetics

## Abstract

Background: Volatile anesthetics were used as sedative agents in COVID-19 (Coronavirus Disease 2019) invasively ventilated patients for their potentially beneficial pharmacological effects and due to the temporary shortages of intravenous agents during the pandemic crisis. Methods: Online databases (PubMed, EMBASE, The Cochrane Central Register of Controlled Trial) and the “clinicaltrials.gov” website were searched for studies reporting the use of isoflurane, sevoflurane or desflurane. Results: We identified three manuscripts describing the beneficial effects of isoflurane on 41 COVID-19 patients with acute respiratory distress syndrome (ARDS) in Germany (*n* = 2) and in the USA (*n* = 1), in terms of reduction in the use of opioids and other sedatives. We also found a case report of two patients with transient nephrogenic diabetes insipidus, which started after 6 and 8 days of sevoflurane sedation. We identified two randomized controlled trials (RCTs; 92 patients overall), two observational studies (238 patients) on the use of volatile anesthetics in COVID-19 patients that were completed but not yet published, and one RCT interrupted for a low recruitment ratio (19 patients) and thus not published. We also identified five ongoing RCTs on the use of inhaled sedation in ARDS, which are also likely to be recruiting COVID-19 patients and which have currently enrolled a total of >1643 patients. Conclusion: Isoflurane was the most frequently used volatile agent in COVID-19 patients and allowed a reduction in the use of other sedative and analgesic drugs. Randomized evidence is building up and will be useful to confirm or challenge these findings.

## 1. Introduction

The COVID-19 pandemic has challenged healthcare systems worldwide since the beginning of 2020, causing a dramatic number of intensive care unit (ICU) admissions. By September 2021, the number of cases worldwide exceeded two hundred and twenty-eight million, with more than four million deaths [1]. Most hospitalized patients with COVID-19 pneumonia have or develop ARDS and a relevant percentage of patients then requires a transfer to an ICU and invasive mechanical ventilation, with an eventual mortality rate in the range of 20–60% [2].

Animal studies demonstrate that volatile anesthetics have beneficial effects on injured lungs in models of ARDS [3]. Multiple biological effects, such as immunomodulatory, anti-inflammatory, and bronchodilator effects, were observed [4,5,6]. Moreover, anti-thrombotic effects and anti-microbial properties were reported [7,8].

Several RCTs performed in an ICU were pooled in a meta-analysis, which suggested a reduced time on mechanical ventilation when using volatile anesthetics in critically ill patients [9]. In particular, the inhalation of both isoflurane and sevoflurane during mechanical ventilation protected against lung injury and suppressed pulmonary inflammatory response [10,11]. Improvements in oxygenation and a decrease in inflammatory markers were reported [12].

Volatile anesthetics in the management of ICU COVID-19 patients were taken into consideration for their potentially beneficial role and due to the massive requirement for intravenous sedatives caused by the international shortage during the pandemic [13,14]. In addition, the unusually high doses of sedation to facilitate ventilator synchrony and the prone position in these patients has justified the use of volatile anesthetics as an alternative or an addition to intravenous sedation [15]. We systematically identified all the literature published and all the currently ongoing trials on volatile anesthetics in COVID-19 patients to enlighten the pros/cons of their role in this setting. 

## 2. Materials and Methods

This systematic review was conducted following the Preferred Reporting Items for Systematic Reviews and Meta-Analyses (PRISMA) guidelines and was submitted for Prospero registration (ID 288553, sent on 31 October 2021) [16]. 

We included only studies with invasively ventilated COVID-19 patients. We considered studies investigating volatile anesthetics (desflurane, isoflurane, sevoflurane): case reports, case series, and comparative studies. We used a “Population, Intervention, Comparator, Outcomes” (PICO) framework for inclusion criteria: invasively ventilated COVID-19 patients admitted to ICU (P), receiving volatile agents (desflurane, isoflurane, sevoflurane) as sedatives (I), eventually compared to intravenous sedation (C) with any reported clinically or laboratory relevant findings (O). 

Two authors independently searched the following databases: PubMed, EMBASE, The Cochrane Central Register of Controlled Trial, and the “clinicaltrials.gov” website, up to November 2021. The databases were searched with the following highly sensitive strategy: (COVID-19 OR Coronavirus OR SARS-CoV-2 OR Severe acute respiratory syndrome coronavirus 2) AND (Isoflurane OR Sevoflurane OR Desflurane OR Helium OR Argon OR Xenon OR Helium OR Nitrous Oxide OR halogenated OR inhalational sedation OR volatile anesthetics).

**Figure 1:**
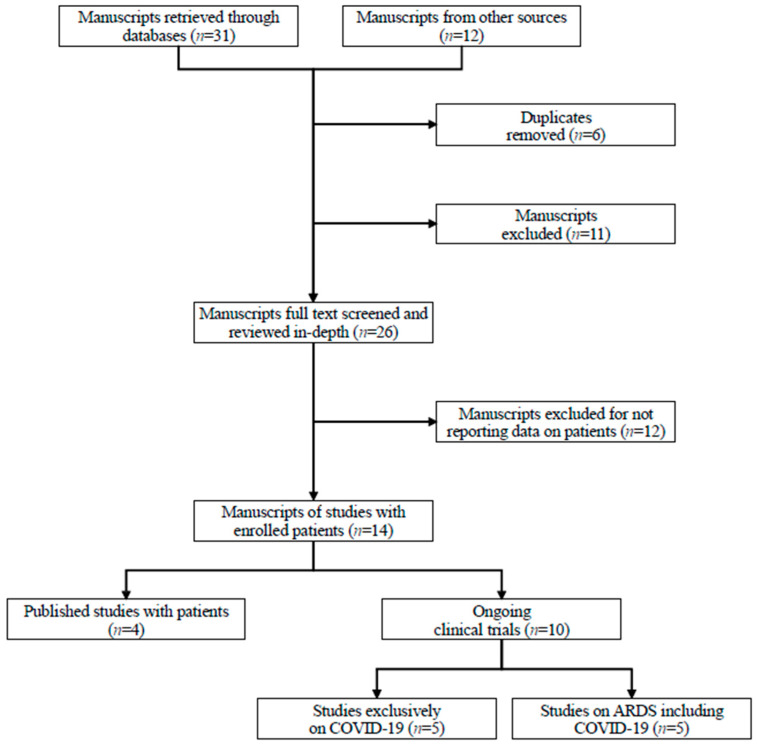
Selection of included studies.

Moreover, the authors used a snowballing strategy to find additional materials and retrieved the references of articles identified by this search strategy selecting the relevant ones. For this review, manuscripts were selected by analyzing titles, abstracts, and the full text if potentially considered relevant (Figure 1). 

After removing duplicates, eligibility assessment and extraction were carried out by the same two authors, independently. When disagreements were present, a third reviewer was involved to resolve them. The search strategy identified mainly clinical studies with data, manuscripts without data, and ongoing clinical trials. Extracted data included the first author, the publication year, the country, the setting, and, if present, the number of patients enrolled. In addition, the agent involved, the delivery device, the reason for and the duration of sedation, the need for extracorporeal membrane oxygenation (ECMO), and the study results were analyzed. When needed, means and standard deviations (SD) were estimated. 

## 3. Results

We identified four manuscripts (Table 1) reporting 43 COVID-19 patients receiving volatile anesthetics (isoflurane *n* = 41, sevoflurane *n* = 2) as sedatives while admitted to an ICU. Different delivery systems were used: the Mirus system (Mirus, TIM GmbH, Koblenz, Germany), the Draeger Apollo device (Draeger Medical, Telford, PA, USA), and the Anaesthetic Conserving Device (AnaConDa, Sedana Medical, Danderyd, Sweden).

A persistent failure to reach the targeted sedation level was the major reason for initiating volatile sedation. Most notably, in two cases, the isoflurane successfully resolved episodes of bronchoconstriction. The mean duration of the volatile sedation ranged from 4.3 to 9.5 days. 

The use of isoflurane was efficient in achieving the targeted level of deep sedation and reduced the needs for propofol, neuromuscular blocking agents (NMBAs), and opioids. Flinspach et al. also observed an improved lung function, in terms of the PaO_2_/FiO_2_ ratio, on the first day of sedation with isoflurane. Hanidziar et al. considered inhaled ICU sedation to be feasible even if it required the continuous presence and vigilance of anesthesia-trained personnel. Kermad et al. administered isoflurane to 20 COVID-19 patients (9 of them on ECMO) and obtained an efficient sedation, while administering fewer NMBAs, less polypharmacy, and lower opioid doses compared to propofol; however, sedation with isoflurane was associated with an increased norepinephrine use. Globally, the feasibility of inhaled sedation for COVID-19 patients undergoing ECMO has been reported in 12 patients [18]. 

No cases of malignant hyperthermia or hepatotoxicity were reported. Coppola et al. reported two cases of transient renal toxicity after a prolonged and continuous administration of sevoflurane (6 and 8 days, respectively). 

We also identified eight unpublished RCTs and two unpublished observational studies (Table 2). The study population includes adult patients with ARDS admitted to an ICU and the intervention drug is either sevoflurane or isoflurane in all studies. To date, three of the eight RCTs have been completed after recruiting 111 overall patients. The two observational studies have been completed with 238 patients enrolled. 

## 4. Discussion 

The most important finding of this systematic review is to demonstrate the feasibility of using volatile anesthetics for the sedation of critically ill, invasively mechanically ventilated COVID-19 patients, including those on ECMO [19]. Volatile anesthetics are a suitable and promising alternative to standard intravenous sedation, as highlighted in several countries that experienced a shortage of intravenous sedative drugs during the COVID-19 pandemic. We also identified several reviews and expert opinion articles discussing in detail the theoretical beneficial effects of using volatile anesthetics for sedation in an ICU setting. Furthermore, we were able to identify several ongoing RCTs comparing volatile anesthetics with propofol in COVID-19 and non-COVID-19 patients in ICU settings. 

Interestingly, several authors have hypothesized that COVID-19 ARDS patients require high doses of sedative drugs [15,20]; the possible underlying reasons for this phenomenon are still unknown, but one of the credible hypotheses is that these patients are generally young and without baseline comorbidities. Furthermore, in some centers, a deep sedation is crucial and repeatedly required for ventilator synchrony, prone positioning, and ECMO therapy. Besides, deep sedation has been speculated to provide optimal patient care, while minimizing the potential aerosol generation from coughing, thus protecting medical personnel [21,22,23].

The use of volatile anesthetics for ICU sedation is already included in national guidelines. According to the German national taskforce guideline, the use of volatile anesthetics is recommended in critically ill patients with ARDS for a moderate-to-deep sedation [24]. More recently, the use of volatile anesthetics was also recommended in ICU COVID-19 patients based on evidence of their rapid on/offset, minimal metabolism with a low risk of organ toxicity, and bronchodilator and anti-inflammatory properties [25]. Recently, isoflurane has been introduced into clinical practice as a valid alternative for ICU sedation in several European countries, following the results from the IsoConDa trial and received national approval from the French Agency for the Safety of Health Products [26,27].

The pharmacologic benefits of volatile anesthetics include a limited systemic accumulation and low levels of hepatic metabolism allowing fast emergence after sedation interruption [28,29]. Moreover, they have bronchodilator effects and antiepileptic properties and have been used successfully in refractory status asthmaticus and status epilepticus [15,30,31]. Indeed, in animal studies, isoflurane has been shown to be a vasodilator in the coronary circulation and to have bronchodilatory effects that might be useful in managing patients with preexisting asthma or chronic obstructive pulmonary disease [3,4,5,6]. 

All this knowledge further supports their use in patients with COVID-19 ARDS. [15,29]

Further benefits of volatile anesthetics in COVID-19 patients might depend on the attenuation of lung inflammation and airways dilation: these effects are mediated by γ-aminobutyric acid type A (GABA_A_) receptors [32,33] and by a decrease in the macrophage release of tumor necrosis factor (TNF)-α, interleukin (IL)-6, IL-1β, Monocyte Chemotactic Protein-1 (MCP-1) and Macrophage Inflammatory Protein-2 (MIP-2). [34] Antithrombotic (sevoflurane enhances P-selectin expression on platelets and increases the binding ability of immune cells) and antimicrobial (in vitro suppression of viral replication) effects have been documented as well [34] (Table 3).

Whereas the use of volatile anesthetics during cardiopulmonary bypass is relatively common, only a few authors have reported it in ECMO patients [15,35,36]. In our systematic review, twelve patients from two different experiences received isoflurane while on ECMO without any technical issues [13,15]. It should also be remembered that hyperlipidemia caused by propofol might be associated with an increased risk of a mechanical failure of the ECMO [37,38]. Interestingly, propofol increases the expression of the angiotensin-converting enzyme 2 (ACE-2) in human pulmonary vessels, which [39] is known as the cell entry receptor; therefore, the use of propofol in these patients has been discouraged [40,41]. However, up to now, there is no correlation between volatile anesthetics and ACE-2 expression. 

The beneficial effect of volatile anesthetics as sedative agents in ICU patients had already been documented in the pre-COVID-19 era [31]. A decreased mortality was observed in a retrospective analysis of long-term, mechanically ventilated patients sedated with isoflurane compared to propofol/midazolam sedation. In a meta-analysis of twelve RCTs with 934 patients, Landoni et al. found that volatile anesthetics reduced the time to extubation in medical and surgical ICU patients [9].

A recent post hoc analysis suggested that volatile anesthetics might have a clinically relevant cardioprotective effect, and they may reduce long-term cardiac mortality [42].

Notwithstanding, the following drawbacks should be mentioned: although its incidence is very low, a history or risk factor of malignant hyperthermia should be investigated before sedation with volatile anesthetics [43]; potential sevoflurane nephrotoxicity (polyuria and diabetes insipidus) after a prolonged use was reported in COVID-19 patients [17] and can be prevented by limiting the duration of administration. 

Devices for inhaled ICU sedation (Sedaconda-ACD, Mirus) are now fully approved and deal with the presence of open circuits in ICUs. Indeed, recently, isoflurane itself was approved for inhaled ICU sedation, using the Sedaconda-ACD device [44]. Due to the presence of active carbon-based reflectors (which recycle up to 90% of the expired gas) and adequate scavenging systems added on the expiratory port of the vent (following manufacturers’ recommendations), these systems are safe overall, in particular with regards to the risk of air pollution/staff exposure [44,45].

The limitations of our systematic review are inherent to the designs of included studies, with no randomized evidence available and limited sample sizes. In fact, only four studies were identified, and future updates will be necessary. Nonetheless, to the best of our knowledge, this is the first systematic review performed on the use of volatile anesthetics for sedation in COVID-19 patients admitted to the ICU, thus providing insights for further studies on this topic. 

Furthermore, it would be interesting to investigate whether there are differences in the cognitive effects of intravenous anesthetics versus inhaled ones. The research on the topic is scarce, and there is no strong evidence on which type of anesthetic-based general anesthesia is favorable [46,47]. Either way, since these effects have a greater impact on elderly patients, the majority of patients who arrive in ICUs, we believe this topic should receive greater attention in future studies on the matter.

Future fields of investigation are the burden on local and global environmental pollution, the assessment of cost [48,49], and the long-term quality of life of patients receiving volatile sedation [50].

## 5. Conclusions

The COVID-19 pandemic increased the awareness of ICU physicians on the possible use of volatile anesthetics in mechanically ventilated COVID-19 patients, including those on ECMO. Administration of isoflurane and sevoflurane is feasible. Obviously, the use of volatile anesthetics requires an appropriate setting and a safe management for exposed healthcare workers. The available evidence seems to support the use of volatile anesthetics in critically ill COVID-19 patients. Ongoing clinical studies will help the scientific community obtain proof of the beneficial effects of volatile anesthetics in providing better patient care in the ICU setting, especially in COVID-19 critically ill patients.

## Figures and Tables

**Table 1:** Manuscripts with data.

Authors	Year	Month	Setting	Patients Enrolled (*n*)	Agent	Comparison Group (*n*)	Delivery System	Reason	Duration of Volatile Sedation (Mean ± SD, Days)	ECMO	Study Results
Coppola S. [17]	2021	April	ICU	2	Sevoflurane	ND	ND	Suboptimal sedation	9.5 ± 0.71	ND	The prolonged use of sevoflurane together with the ARDS related inflammatory and hemodynamic mechanisms on renal function could be nephrotoxic
Flinspach A. [15]	2020	October	ICU	5	Isoflurane	ND	Mirus	3/5: suboptimal sedation 2/5: suboptimal sedation + bronchoconstriction	4.3 ± 3.08	3	Isoflurane achieved the required deep sedation and reduced the need for IV sedation
Hanidziar D. [18]	2021	March	Operating theatre used as ICU	18	Isoflurane + IV sedation	17	Apollo	6/18: as primary sedative during neuromuscular blockade 12/18: as an adjunct to multiple IV agents	5.6 ± 2.99	ND	Isoflurane was associated with a significant decrease in propofol and hydromorphone infusions
Kermad A. [13]	2021	June	ICU	18	Isoflurane ± IV sedation	12	AnaConDa	ND	ND	9	Isoflurane provided sufficient sedation with less NMBAs, less polypharmacy, and lower opioid doses compared to propofol

Abbreviations: ARDS, acute respiratory distress syndrome; ICU, intensive care unit; IV, intravenous; NMBAs, neuromuscular blocking agents; ND, not determined.

**Table 2:** Ongoing clinical trials on the use of halogenated anesthetics in ICU patients with ARDS secondary or not to COVID-19.

Authors	Year	NCT	Patients Enrolled (*n*)	Agent	Comparison Group (*n*)	COVID-19 Patients	RCTs	Recruitment Status	Objective
Blondonnet R.	2020	04023305	43	Sevoflurane	ND	ND	YES	Recruiting	Pharmacokinetic models of sevoflurane-induced sedation during ARDS depending on the lung imaging phenotype.
Fundación para la Investigación del Hospital Clínico de Valencia	2020	04359862	19	Sevoflurane	Propofol	YES	YES	Terminated (low recruitment ratio)	PaO_2_/FiO_2_ on day two in patients with COVID-19 ARDS.
Jabaudon M.	2020	04383730	203	Isoflurane or sevoflurane	Intravenous sedation	YES	NO	Completed	Number of days off the ventilator on day 28.
Jabaudon M.	2020	04235608	700	Sevoflurane	Propofol	ND	YES	Recruiting	Mortality and morbidity.
Jerath A.	2020	04415060	752	Isoflurane or sevoflurane	Propofol	ND	YES	Recruiting	Hospital mortality, ventilator-free days, ICU free days and participant quality of life at 3- and 12-months post discharge.
Lai C.	2020	04530188	68	Sevoflurane	Propofol	ND	YES	Not yet recruiting	PVPI and the amount of EVLW in patients with moderate-to-severe ARDS.
Likhvantsev V.	2019	04014218	80	Sevoflurane	Propofol	ND	YES	ND	28-day mortality.
Palacios Chavarria A.	2020	04998253	24	Sevoflurane	Propofol	YES	YES	Completed	Difference in the oxygenation hypoxic pulmonary vasoconstriction.
Schimmer B.	2020	04355962	68	Sevoflurane	Intravenous sedation	YES	YES	Completed	Mortality and organ dysfunction at day 28.
Xie Z	2020	04492943	35	Isoflurane or sevoflurane	ND	ND	NO	Completed	Survival.

Abbreviations: ARDS, acute respiratory distress syndrome; EVLWi, extravascular lung water index; FiO_2_, fraction of inspired oxygen; ICU, intensive care unit; ND, not determined; PaO_2_, partial pressure of oxygen; PVPI, pulmonary vascular permeability index.

**Table 3:** Protective effects of halogenated anesthetics.

Authors	Year	Month	Agent	Target Cells	Effect	Results
L.M. Stolling [3]	2016	Aug	Sevoflurane, Desflurane, Isoflurane, Halotane	Neutrophil, Macrophage, NK cell, T lymphocyte, B lympohocyte	Decreased cell numbers and cytokine release, Promotion of cell-mediated immunity	The majority of studies reported thus far show that volatile anesthetics have immunosuppressive effects.
D. Suter [4]	2007	Mar	Sevoflurane	Alveolar epithelial cells	Suppression of the expression of inflammatory mediators. These results suggest that sevoflurane reduces AEC-induced accumulation of neutrophils in LPS injury.	This study shows that sevoflurane alters the LPS-induced inflammatory response, not only with respect to the expression pattern of inflammatory mediators, but also regarding the biological consequences with less accumulation of effector cells such as neutrophils.
M. Steurer [5]	2009	Feb	Sevoflurane	Alveolar macrophages	Protective effect of post-conditioning with the volatile anaesthetic sevoflurane on endotoxin-induced injury in AM attenuating cytokine and chemokine production.	Pharmacological post-conditioning prevents enhanced expression of inflammatory mediators and attenuates increased chemotaxis.
S. Voigtsberger [6]	2009	Dec	Sevoflurane	ND	Expression of the cytokine’s protein in bronchoalveolar lavage fluid as well as messenger RNA in lung tissue was significantly lower in the sevoflurane-lipopolysaccharide group compared with the propofol-lipopolysaccharide group.	Significant improvement of the ratio of oxygen tension to inspired oxygen fraction was shown with sevoflurane compared with propofol.
J.N. Harr [7]	2012	Aug	Isoflurane	Platelet	Inhibition of the platelet ADP-pathway	Isoflurane attenuates ALI through an antiplatelet mechanism.
M. Martínez-Serrano [8]	2017	Apr	Sevoflurane, Isoflurane	Against pathogens resistant	Halogenated anaesthetics have shown antibacterial activity.	Both halogenated agents, but particularly isoflurane, showed in vitro antibacterial activity against pathogens resistant to conventional antibiotics.

Abbreviations: AEC, alveolar epithelial cells; ALI, acute lung injury; AM, alveolar macrophages; LPS, lipopolysaccharides; ND, not determined; NK cell, natural killer cel.

## Data Availability

All data generated or analyzed during this study are included in this published article.

## References

[1] Hopkins J. (2021). Coronavirus Resource Center. https://coronavirus.jhu.edu/.

[2] Welker C., Huang J., Gil I., Ramakrishna H. (2022). 2021 Acute Respiratory Distress Syndrome Update, with Coronavirus Disease 2019 Focus. J. Cardiothorac. Vasc. Anesth..

[3] Stollings L.M., Jia L.J., Tang P., Dou H., Lu B., Xu Y. (2016). Immune Modulation by Volatile Anesthetics. Anesthesiology.

[4] Suter D., Spahn D.R., Blumenthal S., Reyes L., Booy C., Z’graggen B.R., Beck-Schimmer B. (2007). The immunomodulatory effect of sevoflurane in endotoxin-injured alveolar epithelial cells. Anesth. Analg..

[5] Steurer M., Schläpfer M., Steurer M., Roth Z’graggen B., Booy C., Reyes L., Spahn D.R., Beck-Schimmer B. (2009). The volatile anaesthetic sevoflurane attenuates lipopolysaccharide-induced injury in alveolar macrophages. Clin. Exp. Immunol..

[6] Voigtsberger S., Lachmann R.A., Leutert A.C., Schläpfer M., Booy C., Reyes L., Urner M., Schild J., Schimmer R.C., Beck-Schimmer B. (2009). Sevoflurane Ameliorates Gas Exchange and Attenuates Lung Damage in Experimental Lipopolysaccharide-induced Lung Injury. Anesthesiology.

[7] Harr J.N., Moore E.E., Stringham J., Wohlauer M.V., Fragoso M., Jones W.L., Gamboni F., Silliman C.C., Banerjee A. (2012). Isoflurane prevents acute lung injury through ADP-mediated platelet inhibition. Surgery.

[8] Martínez-Serrano M., Gerónimo-Pardo M., Martínez-Monsalve A., Crespo-Sánchez M.D. (2017). Antibacterial effect of sevoflurane and isoflurane. Rev. Esp. Quimioter..

[9] Landoni G., Pasin L., Cabrini L., Scandroglio A.M., Baiardo Redaelli M., Votta C.D., Bellandi M., Borghi G., Zangrillo A. (2016). Volatile Agents in Medical and Surgical Intensive Care Units: A Meta-Analysis of Randomized Clinical Trials. J. Cardiothorac. Vasc. Anesth..

[10] Faller S., Strosing K.M., Ryter S.W., Buerkle H., Loop T., Schmidt R., Hoetzel A. (2012). The volatile anesthetic isoflurane prevents ventilator-induced lung injury via phosphoinositide 3-kinase/Akt signaling in mice. Anesth. Analg..

[11] Fortis S., Spieth P.M., Lu W.Y., Parotto M., Haitsma J.J., Slutsky A.S., Zhong N., Mazer C.D., Zhang H. (2012). Effects of anesthetic regimes on inflammatory responses in a rat model of acute lung injury. Intensive Care Med..

[12] Jabaudon M., Boucher P., Imhoff E., Chabanne R., Faure J.S., Roszyk L., Thibault S., Blondonnet R., Clairefond G., Guérin R. (2017). Sevoflurane for Sedation in Acute Respiratory Distress Syndrome. A Randomized Controlled Pilot Study. Am. J. Respir. Crit. Care Med..

[13] Kermad A., Speltz J., Danziger G., Mertke T., Bals R., Volk T., Lepper P.M., Meiser A. (2021). Comparison of isoflurane and propofol sedation in critically ill COVID-19 patients-a retrospective chart review. J. Anesth..

[14] Ferrière N., Bodenes L., Bailly P., L’Her E. (2021). Shortage of anesthetics: Think of inhaled sedation!. J. Crit. Care.

[15] Flinspach A.N., Zacharowski K., Ioanna D., Adam E.H. (2020). Volatile Isoflurane in Critically Ill Coronavirus Disease 2019 Patients-A Case Series and Systematic Review. Crit. Care Explor..

[16] Moher D., Liberati A., Tetzlaff J., Altman D.G. (2009). The PRISMA Group Preferred Reporting Items for Systematic Reviews and Meta-Analyses: The PRISMA Statement. PLoS Med..

[17] Coppola S., Cenci S., Cozzolino M., Chiumello D. (2021). Sevoflurane sedation and nephrogenic diabetes insipidus in patients affected with severe acute respiratory syndrome coronavirus 2. Eur. J. Anaesthesiol..

[18] Hanidziar D., Baldyga K., Ji C.S. (2021). Standard sedation and sedation with isoflurane in mechanically ventilated patients with coronavirus disease 2019. Crit. Care Explor..

[19] Cabibel R., Gerard L., Maiter D., Collin V., Hantson P. (2019). Complete Nephrogenic Diabetes Insipidus After Prolonged Sevoflurane Sedation: A Case Report of 3 Cases. A&A Pract..

[20] Hanidziar D., Bittner E.A. (2020). Sedation of Mechanically Ventilated COVID-19 Patients: Challenges and Special Considerations. Anesth. Analg..

[21] Marini J.J., Gattinoni L. (2020). Management of COVID-19 Respiratory Distress. JAMA.

[22] Meng L., Qiu H., Wan L., Ai Y., Xue Z., Guo Q., Deshpande R., Zhang L., Meng J., Tong C. (2020). Intubation and Ventilation amid the COVID-19 Outbreak: Wuhan’s Experience. Anesthesiology.

[23] Sorbello M., El-Boghdadly K., Di Giacinto I., Cataldo R., Esposito C., Falcetta S., Merli G., Cortese G., Corso R.M., Bressan F. (2020). The Italian coronavirus disease 2019 outbreak: Recommendations from clinical practice. Anaesthesia.

[24] Wolf A., Mörgeli R., Müller A., Weiss B., Spies C. (2017). Delir, Analgesie und Sedierung in der Intensivmedizin: Entwicklung eines protokollbasierten Managements [Delirium, analgesia, and sedation in intensive care medicine: Development of a protocol-based management approach]. Med. Klin. Intensivmed. Notfmed..

[25] ASA/APSF Guidance for Use of Volatile Anesthesic for Sedation of ICU Patients Emergency Use for the COVID-19 Pandemic. (Modified from a protocol produced by Brian O’Gara MD MPH, Assistant Professor of Anesthesia, Beth Israel Deaconess Medical Center, Department of Anesthesia, Critical Care, and Pain Medicine, Harvard Medical School). Am. Soc. Anesthesiol..

[26] Meiser A., Volk T., Wallenborn J., Guenther U., Becher T., Bracht H., Schwarzkopf K., Knafelj R., Faltlhauser A., Thal S.C. (2021). Inhaled isoflurane via the anaesthetic conserving device versus propofol for sedation of invasively ventilated patients in intensive care units in Germany and Slovenia: An open-label, phase 3, randomised controlled, non-inferiority trial. Lancet Respir. Med..

[27] (2021). French Agency for the Safety of Health Products. https://www.emergobyul.com/resources/asnm-french-agency-safety-health-products.

[28] Kim H.Y., Lee J.E., Kim H.Y., Kim J. (2017). Volatile sedation in the intensive care unit: A systematic review and meta-analysis. Medicine.

[29] Jerath A., Ferguson N.D., Cuthbertson B. (2020). Inhalational volatile-based sedation for COVID-19 pneumonia and ARDS. Intensive Care Med..

[30] Jerath A., Panckhurst J., Parotto M., Lightfoot N., Wasowicz M., Ferguson N.D., Steel A., Beattie W.S. (2017). Safety and Efficacy of Volatile Anesthetic Agents Compared with Standard Intravenous Midazolam/Propofol Sedation in Ventilated Critical Care Patients: A Meta-analysis and Systematic Review of Prospective Trials. Anesth. Analg..

[31] Bellgardt M., Bomberg H., Herzog-Niescery J., Dasch B., Vogelsang H., Weber T.P., Steinfort C., Uhl W., Wagenpfeil S., Volk T. (2016). Survival after long-term isoflurane sedation as opposed to intravenous sedation in critically ill surgical patients: Retrospective analysis. Eur. J. Anaesthesiol..

[32] Orser B.A., Wang D.S., Lu W.Y. (2020). Sedating ventilated COVID-19 patients with inhalational anesthetic drugs. EBioMedicine.

[33] Nieuwenhuijs-Moeke G.J., Jainandunsing J.S., Struys M.M.R.F. (2020). Sevoflurane, a sigh of relief in COVID-19?. Br. J. Anaesth..

[34] Suleiman A., Qaswal A.B., Alnouti M., Yousef M., Suleiman B., Jarbeh M.E., Alshawabkeh G., Bsisu I., Santarisi A., Ababneh M. (2021). Sedating Mechanically Ventilated COVID-19 Patients with Volatile Anesthetics: Insights on the Last-Minute Potential Weapons. Sci. Pharm..

[35] Rand A., Zahn P.K., Schildhauer T.A., Waydhas C., Hamsen U. (2018). Inhalative sedation with small tidal volumes under venovenous ECMO. J. Artif. Organs.

[36] Meiser A., Groesdonk H.V., Bonnekessel S., Volk T., Bomberg H. (2018). Inhalation Sedation in Subjects with ARDS Undergoing Continuous Lateral Rotational Therapy. Respir. Care.

[37] Sherren P.B., Ostermann M., Agarwal S., Meadows C.I.S., Ioannou N., Camporota L. (2020). COVID-19-related organ dysfunction and management strategies on the intensive care unit: A narrative review. Br. J. Anaesth..

[38] Son K.H., Lee S.I., Choi C.H., Park C.H. (2017). Mechanical Failure of Extracorporeal Membrane Oxygenation Induced by Hypertriglyceridemia. Ann. Thorac. Surg..

[39] Cao L., Xu L., Huang B., Wu L. (2012). Propofol increases angiotensin-converting enzyme 2 expression in human pulmonary artery endothelial cells. Pharmacology.

[40] Hirota K., Lambert D.G. (2020). Propofol and SARS-CoV-2 infection. Br. J. Anaesth..

[41] Sohn J.T. (2021). Propofol and sedation in patients with coronavirus disease. Am. J. Emerg. Med..

[42] Zangrillo A., Lomivorotov V.V., Pasyuga V.V., Belletti A., Gazivoda G., Monaco F., Neto C.N., Likhvantsev V.V., Bradic N., Lozovskiy A. (2022). Effect of Volatile Anesthetics on Myocardial Infarction After Coronary Artery Surgery: A Post Hoc Analysis of a Randomized Trial. J. Cardiothorac. Vasc. Anesth..

[43] Kaura V., Hopkins P.M. (2020). Sevoflurane may not be a complete sigh of relief in COVID-19. Br. J. Anaesth..

[44] Sackey P.V., Martling C.R., Nise G., Radell P.J. (2005). Ambient isoflurane pollution and isoflurane consumption during intensive care unit sedation with the Anesthetic Conserving Device. Crit. Care Med..

[45] González-Rodríguez R., Muñoz Martínez A., Galan Serrano J., Moral García M.V. (2014). Health worker exposure risk during inhalation sedation with sevoflurane using the (AnaConDa^®^) anaesthetic conserving device. Rev. Esp. Anestesiol. Reanim..

[46] Zhang Y., Shan G.J., Zhang Y.X., Cao S.-J., Zhu S.-N., Li H.-J., Ma D., Wang D.-X. (2018). Propofol compared with sevoflurane general anaesthesia is associated with decreased delayed neurocognitive recovery in older adults. Br. J. Anaesth..

[47] Li Y., Chen D., Wang H., Wang Z., Song F., Li H., Ling L., Shen Z., Hu C., Peng J. (2021). Intravenous versus Volatile Anesthetic Effects on Postoperative Cognition in Elderly Patients Undergoing Laparoscopic Abdominal Surgery. Anesthesiology.

[48] Charlesworth M., Swinton F. (2017). Anaesthetic gases, climate change, and sustainable practice. Lancet Planet. Health.

[49] Boldt J., Jaun N., Kumle B., Heck M., Mund K. (1998). Economic considerations of the use of new anesthetics: A comparison of propofol, sevoflurane, desflurane, and isoflurane. Anesth. Analg..

[50] Chen C., Ji M., Xu Q., Zhang Y., Sun Q., Liu J., Zhu S., Li W. (2015). Sevoflurane attenuates stress-enhanced fear learning by regulating hippocampal BDNF expression and Akt/GSK-3β signaling pathway in a rat model of post-traumatic stress disorder. J. Anesth..

